# Pressure modulates the self-cleavage step of the hairpin ribozyme

**DOI:** 10.1038/ncomms14661

**Published:** 2017-03-30

**Authors:** Caroline Schuabb, Narendra Kumar, Salome Pataraia, Dominik Marx, Roland Winter

**Affiliations:** 1 Physikalische Chemie I—Biophysikalische Chemie, Fakultät für Chemie und Chemische Biologie, TU Dortmund, Otto-Hahn-Strasse 4a, Dortmund 44227, Germany; 2 Lehrstuhl für Theoretische Chemie, Ruhr-Universität Bochum, Bochum 44780, Germany

## Abstract

The ability of certain RNAs, denoted as ribozymes, to not only store genetic information but also catalyse chemical reactions gave support to the RNA world hypothesis as a putative step in the development of early life on Earth. This, however, might have evolved under extreme environmental conditions, including the deep sea with pressures in the kbar regime. Here we study pressure-induced effects on the self-cleavage of hairpin ribozyme by following structural changes in real-time. Our results suggest that compression of the ribozyme leads to an accelerated transesterification reaction, being the self-cleavage step, although the overall process is retarded in the high-pressure regime. The results reveal that favourable interactions between the reaction site and neighbouring nucleobases are strengthened under pressure, resulting therefore in an accelerated self-cleavage step upon compression. These results suggest that properly engineered ribozymes may also act as piezophilic biocatalysts in addition to their hitherto known properties.

## Abstract (web summary)

Studying the reactivity of ribozymes under extreme pressure could provide insights to optimize biocatalytic RNA design. Here, the authors show that at high pressure the transesterification step of the hairpin ribozyme self-cleavage reaction accelerates, while the overall process is slower.

Although RNAs have been believed for a long time to be passive genetic blueprints, it is now widely recognized that some RNA molecules—dubbed ribozymes—feature intrinsic enzyme-like activities^1^,^2^. Next to well-characterized small ribozymes that simply cleave phosphodiester bonds, RNA catalysis is known to affect a vast number of cellular reactions. The hairpin ribozyme (HpRz) is one of the small nucleolytic ribozymes able to catalyse reversible sequence-specific cleavage by transesterification within the same molecule^3^. This self-cleavage reaction involves the nucleophilic attack of the 2′-OH group on the adjacent phosphorus atom, resulting in a 2′,3′-cyclic phosphate and a 5′-hydroxyl terminus^4^. Discovery of such catalytically active RNAs, which simultaneously can store genetic information and act as biocatalysts, greatly supported the so-called ‘RNA world' hypothesis, suggesting that extant life arose from molecular precursors where RNA could not only self-replicate but also ensure proper metabolism.

Studies of pressure effects on ribozymatic catalysis are of particular interest in this respect. On the one hand, such studies enable key insights into the catalytic mechanism at stresses encountered in abyssal water, including the hydrothermal vents and volcanic environments proposed to be the birthplace of life^5^,^6^,^7^, where kbar pressures are easily encountered. On the other hand, pressure is an important physical control parameter in addition to temperature that can be used to determine thermodynamic and kinetic parameters, which are otherwise inaccessible^8^,^9^,^10^,^11^,^12^. Furthermore, a particularly promising field is the use of high hydrostatic pressure (HHP) to modulate enzymatic conversions^13^,^14^,^15^. Given that the rate of an enzymatic reaction is often limited by the thermostability of the enzyme, superimposing pressure-induced thermostabilization of the enzyme with an accelerated substrate conversion at increased temperatures can lead to an improved overall reaction rate. Moreover, catalytic activity can directly be enhanced by HHP if the activation volume that is associated with the reaction is negative. Although pressure effects on protein-based enzymes have been extensively studied in the past^9^,^14^,^16^, there are only a few studies where such pressure effects on catalytic reactions have been investigated in the case of ribozymes^17^,^18^,^19^. In particular, detailed analyses of pressure-induced effects on the self-cleavage reaction step of the overall self-cleavage process as catalysed by ribozymes, including the covalent transesterification reaction as well as the various other non-covalent steps of the process such as docking/undocking and dissociation of the product driven by conformational effects, are still largely unexplored. In addition, no attempt has been made so far to invoke computational studies to yield complementary mechanistic information on ribozymatic self-cleavage at HHP conditions.

The particular wild-type hairpin ribozyme (wt-HpRz) under consideration here consists of two independently folding domains, A and B, each consisting of an internal loop and two helices (Fig. 1a). The overall reaction process contains at least three major steps, namely docking of the loops, the chemical cleavage step itself and finally undocking/dissociation of the product (Fig. 1b)^20^,^21^,^22^,^23^,^24^. The docked state, also called the pre-catalytic state being necessary for the catalytic step to take place, is formed by a rearrangement of loops A and B (Fig. 1b) by coming into close vicinity^25^,^26^,^27^. Importantly, it has been found that the neighbouring nucleobases A38 and G8 in the reaction site have important roles in stabilizing the transition state via distinct hydrogen bonding interactions^28^,^29^,^30^,^31^,^32^.

We used state-of-the-art high-pressure static as well as stopped-flow fluorescence resonance energy transfer (FRET) set-ups to carefully characterize different reaction steps^26^ in the complicated ribozyme catalysis reaction process with a special focus on the self-cleavage step. To complement our experiments, we explored the self-cleavage step by simulating the activated precursor (AP) state^33^ being the HpRz structure closest to the transition state (see below for more details), using large-scale replica-exchange molecular dynamics simulations at HHP conditions. Fortunately, we could build on a rich available literature on force field molecular dynamics simulations of RNA or ribozymes at ambient conditions as documented, for instance, in refs 34, 35, 36 among many other works. Importantly, recently improved force fields^37^,^38^ have enabled fairly reliable such simulations up to long time scales^39^. In the realm of HHP conditions, computer simulations have revealed most recently that high pressures stabilize the A-form of RNA folds^40^. In addition to such non-reactive simulations, there has also been impressive progress on QM/MM studies focusing on the very chemical reaction of ribozymes at ambient conditions, see for instance refs 41, 42, 43.

In the present study, we unexpectedly find that although the overall ribozymatic process is known to slow down under pressure^17^, the self-cleavage chemical reaction itself is accelerated. The proposed mechanistic explanation is based on the well-known fact that hydrogen bonding of the reaction site with neighbouring nucleobases has a central role at ambient conditions in establishing the trigonal bipyramidal structure that is essential for the chemical reaction step to occur subsequently^30^,^32^,^33^. Here we find that exactly the same hydrogen bonding interactions that prepare the ribozyme for the self-cleavage reaction to set in get even more enhanced under high-pressure conditions, which we propose to explain at the molecular level the experimentally observed pressure-induced acceleration of the self-cleavage reaction in the compressed HpRz. Hence, given a suitable molecular architecture and appropriate environmental conditions such as ionic strength that favour the conformational non-covalent steps before and after the covalent reaction as sketched in Fig. 1b, high-pressure conditions may, therefore, also accelerate the overall self-cleavage process of ribozymes.

## Results

### Pressure-induced effects on the overall catalytic process

The effect of hydrostatic pressure on the overall self-cleavage process of the HpRz has been carefully investigated previously^17^,^18^,^19^,^44^. Using PAGE (urea denaturing polyacrylamide gel electrophoresis), it has been shown repeatedly that the overall cleavage reaction is retarded upon pressurization^17^,^18^,^19^,^44^. This is the basis of the currently accepted understanding that self-cleavage of present-day ribozymes is suppressed at high pressures.

**Figure 1:**
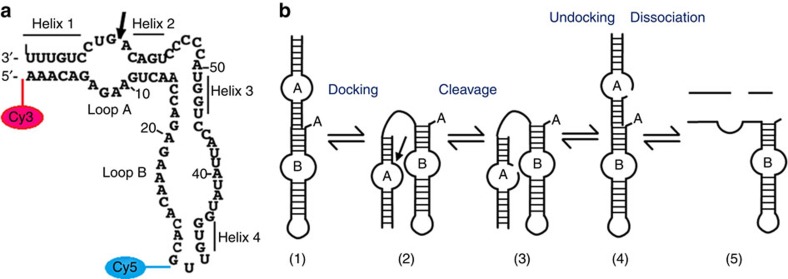
Hairpin ribozyme and schematic representation of different stages in its catalysis. (**a**) Secondary structure of the FRET-labelled minimal wild-type self-cleaving hairpin ribozyme (HpRz) from tobacco ringspot virus satellite. The self-cleavage site is marked by an arrow^25^. (**b**) Schematic representation (scheme based on refs 23, 26) of the entire multistep self-cleavage process of HpRz involving non-covalent steps before and after the covalent self-cleavage reaction (that is, transesterification of the phosphodiester bond) from (2) to (3) as detailed in Fig. 2; note that all structures might still consist of different conformational substates, and all steps have been found to be reversible.

Indeed, our PAGE measurements on wt-HpRz as shown in Fig. 3a confirm that the overall process gets decelerated upon increasing pressure (for experimental details, see Supplementary Note 1). From the marked decrease of the equilibrium self-cleavage reaction at high pressures, using dln*K*/d*p*=−Δ*V*/(*RT*), a volume change of the overall reaction (that is, steps 1–5 in Fig. 1b) of Δ*V*≈+6 ml mol^−1^ can be deduced, where *K* is the apparent equilibrium constant of the whole process. A similar scenario is observed for our mod-HpRz system (Supplementary Fig. 1), including in addition conditions where Mg^2+^ has been replaced by cobalt(III)hexammine ([Co(NH_3_)_6_]^3+^) (for the PAGE images and self-cleavage control measurements, see Supplementary Fig. 2). This is exactly the system for which our detailed molecular dynamics simulations have been carried out (see below). However, the pressure responses of the various steps of the entire cleavage process according to Fig. 1b, including the key transesterification reaction from (2) to (3), are unknown and, therefore, need attention.

### FRET spectroscopy

In order to understand the different steps in the catalysis reaction under pressure, we used different instrumentation. For short time scales, allowing to observe the initial folding (the docking) of the HpRz, a high-pressure stopped-flow (HPSF) apparatus with ms time resolution and fluorescence detection was used. Longer times were recorded using a static high-pressure cell placed into the fluorescence spectrometer set-up (Fig. 3b). As the rate constants for docking, undocking and cleavage increase rapidly with temperature, we used a temperature of 10 °C for the pressure-dependent studies in order to still being able to resolve details of the reaction scheme. Pressure effects up to at least 2 kbar are fully reversible^17^,^19^. The incorporation of fluorescent labels at specific sites of the HpRz (donor dye Cy3 attached at loop A, acceptor dye Cy5 attached in close vicinity to loop B according to Fig. 1a) provides an excellent tool to monitor in real time structural transitions between folded and unfolded states, allowing us to look into the cleavage reaction in more detail^23^. The time trace of the FRET signal of Cy5, which decays rapidly with the separation of the acceptor and donor fluorophores, was fitted to exponential functions, yielding observed apparent rate constants *k*_obs_=1/*τ* (*τ*, characteristic time constant). Also, when the overall equilibrium shifts to the cleaved product, a decrease of the static FRET signal is expected.

### Self-cleavage under pressure

For the cleavage to take place, the formation of the docked state is necessary, which is, therefore, also called the pre-catalytic state. The mechanism that drives the rearrangement of the loops such that the docked state is formed remains largely unknown and remains a matter of study. It has been found that it depends sensitively on the environmental conditions such as temperature, pressure or ionic strength. After the docked state is formed, we followed in real time the effect of pressure on the cleavage kinetics by using a static high-pressure FRET spectroscopy set-up (see the data sets in Fig. 3b starting at 500 s after the docked state has been formed as detected by stopped-flow techniques as detailed in the next section).

Surprisingly, our data indicate that application of 1 and 1.5 kbar pressures increases self-cleavage of HpRz significantly (Fig. 3b and Supplementary Fig. 3). Fitting an exponential curve to the fast decaying FRET signals yields observed rate constants, *k*_obs_, of about 0.6–0.8 min^−1^ compared with the ambient pressure value of 0.03–0.05 min^−1^. This finding unveils that *k*_obs_ increases by more than one order of magnitude upon compression of HpRz into the kbar regime! As *k*_obs_ may generally comprise contributions from cleavage and undocking/dissociation, the latter contributions requiring additional hydration leading to an increase of volume, we may infer that the cleavage step itself is accelerated upon compression, that is, is connected to a negative activation volume. Pressure-induced changes in reaction-competent conformational equilibria between different substates may contribute to the observed changes as well. Moreover, the overall equilibrium between docked and cleaved states is shifted to the docked state compared with ambient pressure as indicated by the increase in the level of equilibrium FRET intensity with rising pressure. This implies that cleavage can hardly be detected anymore by FRET above about 2 kbar (owing to an insufficient signal-to-noise level in our high-pressure sample cell environment when the population of docked states is too low), which is in agreement with the PAGE data from Fig. 3a. From the pressure dependence of the initial decay of the FRET signal and hence the kinetic constant, (dln*k*/d*p*)_*T*_=−Δ*V*^#^/(*RT*), the activation volume of the cleavage/undocking step, Δ*V*^≠^, can be determined, which is negative and amounts to −63±10 ml mol^−1^. These data indicate that the increased pressure induces conformational changes that favour the cleavage/undocking step upon compression.

Moreover, as a control, we studied the pressure effect on the cleavage rate of HpRz in a different setup by applying fast pressure-jumps (for example, of 1 kbar) at different time points of the ambient pressure reaction (Supplementary Fig. 4). Similar to the previous measurements (Fig. 3b), a pressure jump of 1 kbar resulted in a rapid increase of the FRET intensity. This corresponds to an increased docking of the uncleaved ribozyme, which in turn is followed by a more rapid cleavage/unbinding step of those extra docked HpRz molecules compared with the FRET intensity decay at ambient pressure.

### Quantifying pressure effects on the docking step

To recover also the docking step, HPSF data were recorded (as depicted in Fig. 3b up to 500 s from where on the stopped-flow FRET set-up was also used to probe the kinetics of the cleavage step itself once the docked state was formed). The measurement was started from the moment HpRz came into contact with Mg^2+^ ions, up to 500 s. The rate of the apparent kinetic constant, which is expected to be essentially due to the rather slow docking process^23^, that is, *k*_obs_≈*k*_dock_*+k*_undock_, as revealed by the increasing FRET signal amounts to ∼0.4 min^−1^ at ambient pressure (at *T*=10 °C), in good agreement with literature data for the rate constant of docking^23^. The higher FRET intensity observed at 1–2 kbar pressures is a signature of a rapid shift of the docking/undocking equilibrium to some docked state(s). The initial minor decrease and subsequent increase of FRET intensity within the first 500 s at 1–2 kbar pressures might reflect partial cleavage and docking to some different, possibly Mg^2+^-bound, docked state, respectively (see also Supplementary Figs 2 and 3), which occur on a similar time scale. This latter docking phase seems to be retarded at high pressures (rate constants: ∼0.4 min^−1^ at 1 bar, ∼0.2 min^−1^ at 1 kbar), indicating a positive activation volume for docking, thereby contributing to the overall slowing down of the process upon compression.

### Preferred attack arrangement and active region

**Figure 2:**
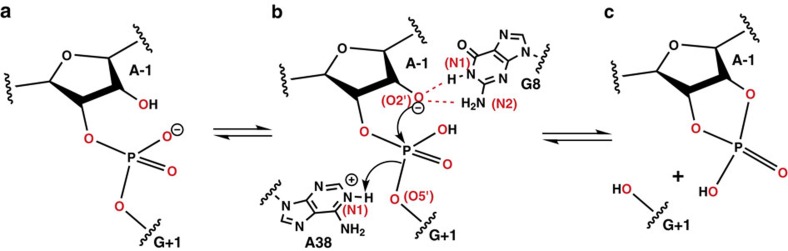
Reaction scheme for the self-cleavage reaction step of the hairpin ribozyme. The activated precursor (AP) state of the ribozyme corresponds to (**b**), where the interacting nucleobases G8 and A38 are also shown. Panels (**a**) and (**c**) correspond to the pre-catalytic state and the products formed, respectively.

**Figure 3:**
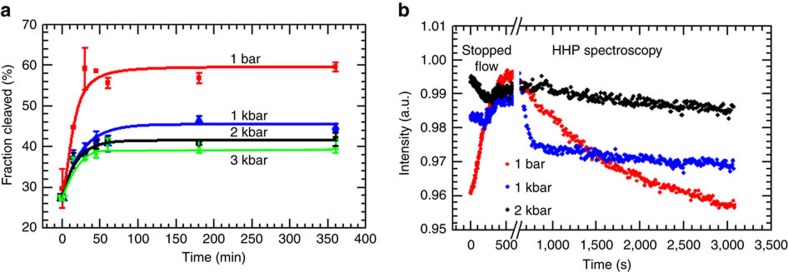
Pressure dependence of the self-cleavage reaction. (**a**) Time evolution of 3.6 μM wt-HpRz self-cleavage reaction (fraction of cleaved RNA after addition of 6 mM Mg^2+^) at different pressures: ambient pressure (1 bar), 1, 2 and 3 kbar (1 kbar=100 MPa). Solution conditions: 50 mM Tris-HCl buffer, 0.1 mM EDTA, pH 7.5, 6 mM MgCl_2_, *T*=10 °C. Error bars represent standard deviation of three independent measurements. (**b**) Effect of pressure on the docking/cleavage reaction, combining the rapid stopped-flow (up to 550 s) and slower (from 600 up to 3,000 s) kinetic FRET data of the Cy3/Cy5 fluorescently labelled HpRz at ambient pressure (red), 1 kbar (blue) and 2 kbar (black). The initial rise of the FRET signal is due to the shift of the docking-undocking equilibrium of the uncleaved ribozyme to the docked state, whereas the subsequent decay is due to the cleavage reaction and undocking/dissociation.

When the ribozyme is in the docked state, the proton from the 2′-OH group is transferred to the scissile phosphate (possibly via water^45^,^46^) as indicated in the step from panel a to b of Fig. 2. The thereby generated 2′-oxygen nucleophile in panel b is stabilized by accepting two H-bonds (indicated by the dotted red lines) from the vicinal G8 nucleobase (shown only in panel b)^30^,^47^. This particular state is the aforementioned AP state^33^. When HpRz is in the AP state, the O2′ nucleophile is prepared to readily attack the scissile phosphate; the O5′ leaving group takes a proton from the already protonated A38 nucleobase at its N1 position as symbolized in panels b to c. The cyclic 2′,3′-phosphate and the 5′-hydroxyl group at G+1 are formed as products of self-cleavage, see panel c.

**Figure 4:**
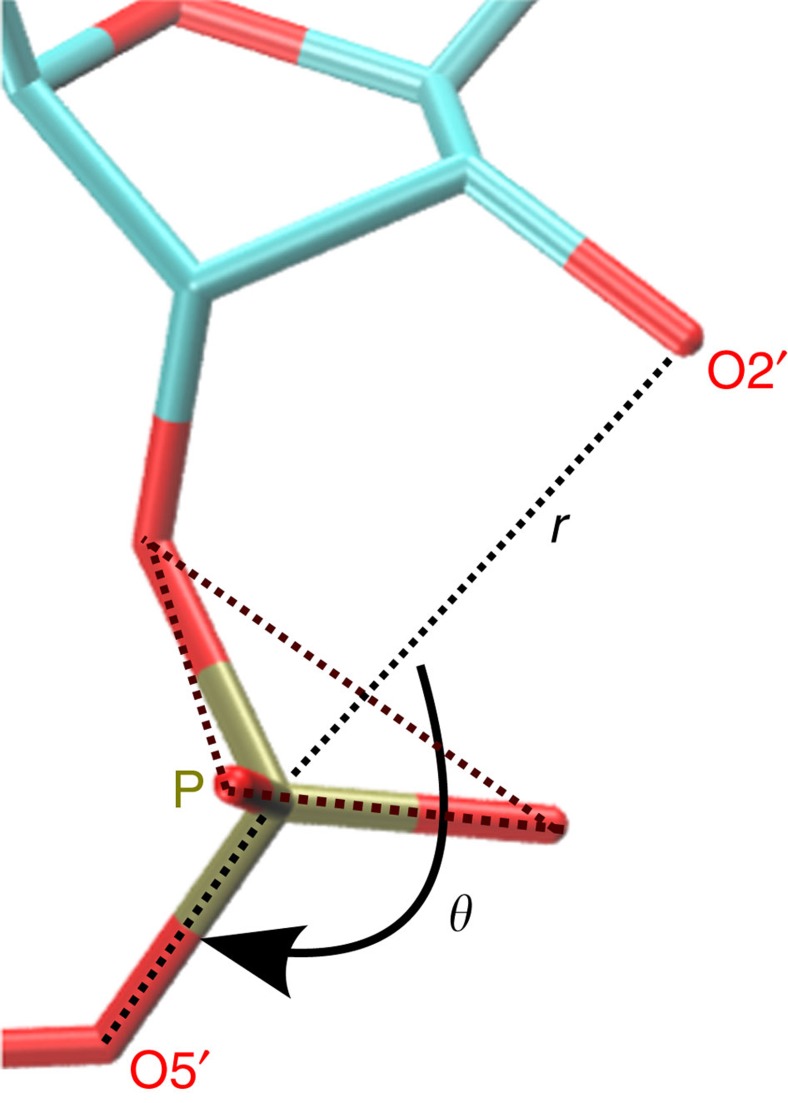
An in-line configuration is required for the cleavage reaction. Preferential attack configuration in the activated precursor (AP) state as depicted schematically in Fig. 2b; *θ* and *r* are the O2'···P···O5' angle and the O2'···P distance, respectively. For an efficient self-cleavage reaction to occur, the attacking O2' site of A-1 should be in-line with respect to the scissile phosphate (P) and the O5' leaving group, whereas the O2'···P distance should be close enough to eventually allow for the formation of the nascent O2'-P covalent bond.

For cleavage of the P-O5′ bond to occur via nucleophilic attack of O2′ on the scissile phosphate (Fig. 2b), the angle between the O2′ nucleophile, the scissile phosphate (P), and the O5′ leaving group must be close to the in-line arrangement, that is, *θ=*180°. Moreover, the O2′ to P distance *r* must be small to eventually lead to O2'-P covalent bond formation^48^. Altogether, this defines so-called ‘active configurations' within the AP state. Thus, the key molecular variables that describe the biocatalytic reaction step itself are *θ* and *r* as defined in Fig. 4 as analysed in detail previously at ambient conditions^33^.

**Figure 5:**
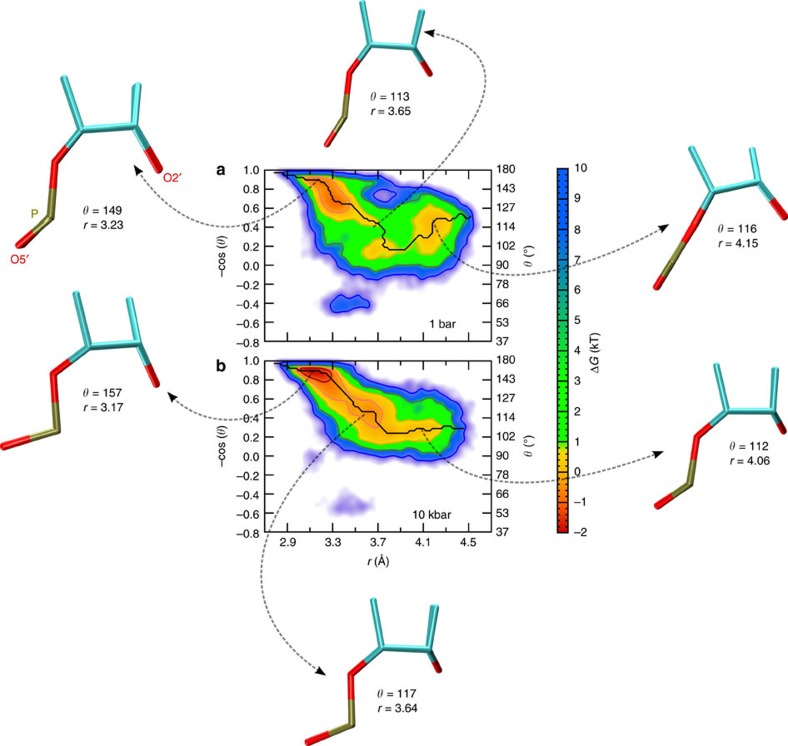
Gibbs free-energy landscapes of the activated precursor state AP of mod-HpRz. (**a**) 1 bar and (**b**) 10 kbar at 300 K in the two-dimensional subspace spanned by the negative cosine of the attack angle -cos(*θ*) (the approximate *θ* scale is reported on the right axis) and the attack distance *r* as defined in Fig. 4; the free energies are reported relative to the inactive region. The presented structural fragments (see Fig. 4) together with the provided angles and distances in **a**,**b** have been obtained by averaging the configurations corresponding to the three relevant regions along the minimum free-energy pathways (see Fig. 6) that are referenced by using dotted arrows in the free-energy surfaces.

Based on these considerations, we have sampled the Gibbs free-energy landscapes of AP at different pressures in the two-dimensional subspace spanned by the *θ* and *r* variables, see Fig. 5a,b. In that plane, we are able to define a region of ‘active configurations' on the free-energy landscapes (Fig. 5) using a half-circular separatrix that approximately separates the global minimum in the upper-left corner of both panels, where *θ* and *r* correspond to attack arrangements that favour the self-cleavage reaction step, from the rest of the free-energy landscape which we call the ‘inactive region'. In order to reveal HHP effects in comparison to ambient conditions, we use a pressure of 10 kbar with the aim to enhance the overall small structural and energetic effects of compression, being well aware that the experiments cannot be performed at pressures exceeding about 2 kbar for the aforementioned sensitivity reasons.

### Free energies and probability distributions

The free-energy landscapes were constructed by properly reweighting the replica exchange data using the multistate Bennett acceptance ratio estimator approach as encoded in the pymbar software^49^, which overcomes the limitations of multiple histogram techniques such as weighted histogram analysis (WHAM)^49^. Multistate Bennett acceptance ratio estimator does not use the discretization of the sampled energy range to produce histograms hence eliminating the bias due to binning of energy. The generated free-energy landscapes were smoothened using a normalized Gaussian kernel with a s.d. of 1.4. The resulting free-energy surfaces are shown in the units of the thermal energy at 300 K, *k*_B_*T*, and referred relative to the local free-energy minima in the inactive region (Fig. 5). Using the two-dimensional probability distribution function, which is given by the exponential of the negative free energy in *k*_B_*T* units, the percentage of configurations in the aforementioned active region was calculated. This procedure leads to roughly 57% of active configurations at 1 bar that is increased to about 70% at 10 kbar; it is noted in passing that the increase of active configurations upon compression is observed independently of varying the separatrix definition within reasonable bounds.

### Pressure effects on free-energy landscapes and reaction pathways

Lower free energies imply that the system is more stable in that region of parameter space compared with other regions. This will lead to an increased population of that subspace thus enhancing the probability to find the system in the associated functional state. In the present context, the free energy in the upper-left region of Fig. 5, corresponding to active (*θ, r*) configurations of the AP state, is clearly lower than in the inactive region. Moreover, the free energy minimum in the active region is found to shift even more towards –cos(*θ*)≈1 (or *θ*≈180°) upon compression and thereby furthermore enhances the favourable in-line attack arrangement. This finding is both visualized and quantitatively supported by the averaged structures shown as insets in Fig. 5 along the pathway where *θ* is found to systematically increase and *r* to decrease in the active region as a result of compression.

**Figure 6:**
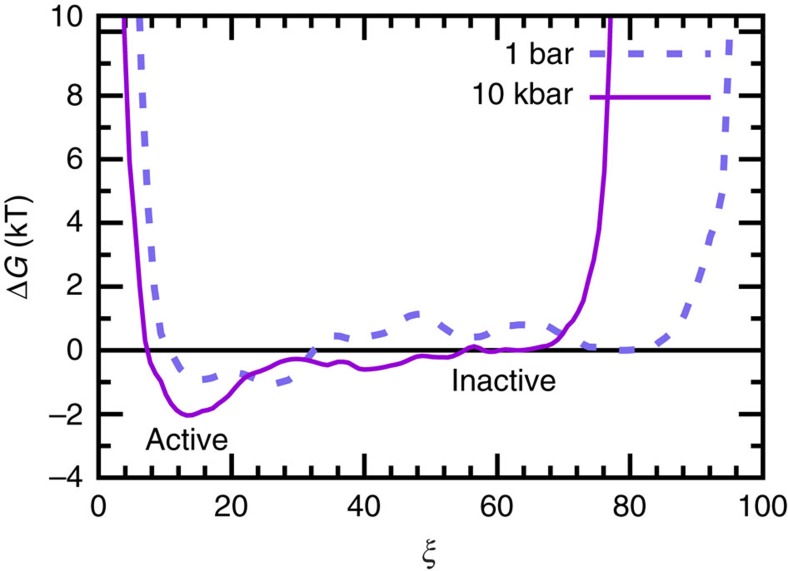
Minimum free-energy pathways connecting active and inactive configurations. Minimum free-energy pathways connecting the active to the inactive basin of AP configurations according to the landscapes in Fig. 5a,b corresponding to ambient and high pressure at 300 K, respectively; free energies are reported relative to the inactive region.

All this indicates that the ribozyme in the active region of the AP state gets stabilized at HHP conditions relative to those configurations that define the inactive region. Therefore, the system will stay longer in the active region prone to attack, thus leading to an increased *a priori* probability for the self-cleavage reaction to proceed under pressure. This is furthermore supported by the finding that the weight of the conformations in the active region of the free-energy landscape increases systematically under pressure. Indeed, along the minimum free-energy pathway (Fig. 6), we can clearly see that the active region is more stabilized relative to the inactive region when applying pressure. Moreover, the free-energy barrier to move from the inactive to the active region is decreased upon compression. Therefore, our simulation results indicate that high pressures favour the self-cleavage step of the mod-HpRz both thermodynamically and kinetically in substantial agreement with our experimental data.

### Pressure-enhanced H-bond network in the self-cleavage site

At this stage, we can use our simulations to disclose the molecular underpinnings of the acceleration phenomenon found in terms of pressure-induced free-energy changes. This is achieved by unraveling the pressure effect on the H-bond network that is known to critically involve the G8 and A38 nucleobases (Fig. 2b) at ambient conditions^32^,^33^,^48^,^50^. Indeed, the crystal structure of the docked state (being the conformational state (2) of Fig. 1b) reveals that the G8 and A38 nucleobases are situated in the close proximity of the scissile phosphate (see Fig. 2b), which structurally connects to the key role they have in the catalytic reaction step itself^29^,^32^. In particular, experimental studies found that A38 is protonated at the N1 position (as depicted in Fig. 2b)^28^,^29^,^48^. On the other hand, G8 acts as a general base, however, it remains in its neutral, deprotonated form^31^. Previously, a QM/MM simulation study of HpRz suggested that G8 rather has a structural role than as a base to activate the O2′ nucleophile^30^. The proton at the N1 nitrogen of A38 forms an H-bond to O5′ of G+1, and thereby supports cleavage of the P-O5' bond. In addition, G8 stabilizes the negative charge on the O2′ site of A-1 by donating one or two H-bonds via N1 and N2, thus helping to maintain the trigonal bipyramidal structure and preparing the nucleophilic attack of O2' at P (Fig. 2b).

**Figure 7:**
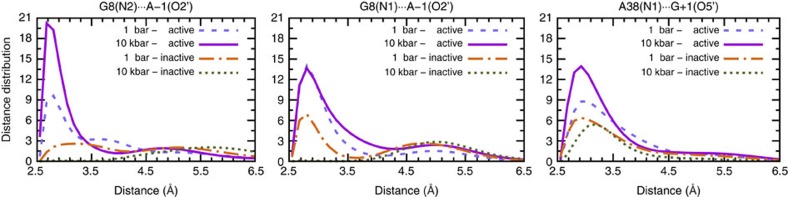
Pressure enhances the H-bond interactions of G8 and A38. Probability distribution functions of relevant interatomic distances (see Fig. 2b) involving H-bond donors of the G8 and A38 nucleobases and the attacking as well as leaving groups of the transesterification cleavage reaction being the H-bond acceptors (that is, O2' and O5') separated into active and inactive configurations of the AP state (see text) at 1 bar and 10 kbar at 300 K. Stabilizing H-bond interactions occur for distances of roughly 2.8 Å, which leads into maintaining the in-line configuration.

Based on these structural insights known from previous work at ambient conditions, we set out to analyse in detail the corresponding G8(N1)···A-1(O2′), G8(N1)···A-1(O2′), and A38(N1)···G+1(O5′) distance probability distribution functions separately for the configurations in the active and inactive regions. The HHP data contrasted to ambient conditions as compiled in Fig. 7 make clear that these stabilizing H-bonds around the scissile phosphate get enhanced in the active region upon compression (as evidenced by the increasing probability of H-bonding contacts at roughly 2.8 Å), whereas the opposite trend is observed in the inactive region. This molecular-level analysis reveals the key role of G8 and A38 in stabilizing the AP state in its active region via an H-bonding network, which is enhanced upon compression and, therefore, results in more favourable conditions for nucleophilic attack and thus faster transesterification of HpRz at high pressures in concert with our experimental findings for that step.

## Discussion

Our study unravels that even though increasing pressures slow down the overall rate of the complex self-cleavage process of the HpRz, which is well-established based on previous work, the biocatalytic transesterification step itself gets accelerated at high pressures. At the molecular level, this surprising finding is traced back to specific H-bonds that are donated by the G8 and A38 nucleobases to the active site, which systematically strengthen upon increasing pressure. These non-covalent interactions act as a scaffold that keeps important atoms around the scissile phosphate in a perfect arrangement for the elementary chemical reaction to occur, hence, increasing the rate of the self-cleavage step at high pressures. Thus, it would be interesting to carry out electronic structure based reactive simulations of HpRz at HHP conditions using the well-established QM/MM approach to the self-cleavage reaction of ribozymes that has been developed for ambient conditions^30^,^34^,^41^,^42^,^43^,^47^. In addition to such refined computer simulations, FTIR difference spectroscopy might offer an interesting experimental avenue to explore the hitherto unknown pressure-induced enhancement of the H-bond network at the cleavage site by extending work done on detecting such network changes inside proteins at work^51^,^52^. Finally, exploring the role of water molecules in compressed ribozymes compared with what is known from ambient conditions^45^,^46^ will provide further molecular insights into the pressure response of these RNA-based enzymes. From the experimental point of view, complementary single-molecule FRET studies along with mutations of sensitive residues may help unraveling further details of the complex multiphasic reaction kinetics of this intricate nucleic acid machinery. The high-pressure set-up for such studies has still to be developed, however.

Beyond the specific case, our results support the perspective that pressure compresses the structure of the docked state of the ribozyme such that the key enzymatic reaction step gets enhanced. We, therefore, may conclude that understanding how RNA functions under extreme conditions could potentially provide novel insights into its function under physiological or optimal conditions, or provide potential design principles for engineered RNAs. Last but not least, our finding is an encouragement to explore intensely the pressure response of ribozymes, which promises to be even richer than that known from the usual protein-based enzymes.

## Methods

### RNA nucleotides

The wt-HpRz (sequence: 5′-AAACAGAGAAGUCAACCAGAGAAACACACGUUGUGGUAUAUUACCUGGUA CCCCCUGACAGUCCUGUUU-3′), FRET-labelled wt-HpRz (FRET-HpRz sequence: 5′-cyanine 3-AAACAGAGAAGUCAACCAGAGAAACACACG-cyanine 5-UUGUGGUAUAUUACCUGGUACCCCCUGACAGUCCUGUUU-3′) and FRET-labelled modified HpRz (mod-HpRz sequence: 5′-cyanine 5-ACGGUGAGAAGGGAGGCAGAGAAACACAC-cyanine 3-GUCGUGGUACAUUACCUGCCACCCCCUCCCAGUCCACCGU-3′; see below for details) molecules were synthesized by IBA Life Solutions GmbH (Goettingen, Germany). These RNA sequences represent the minimal two-way junction model systems of the original four-way junction tobacco ringspot virus satellite RNA. The FRET-HpRz and mod-HpRz molecules have attached two fluorescent dyes, cyanine 3 phosphoramidite (Cy3) and cyanine 5 phosphoramidite (Cy5) to the loop A and at the loop B, respectively, as depicted schematically in Fig. 1a. Their chemical nature is built from two indole rings that are connected by a polymethine chain. Cy3 serves as the donor fluorophore and Cy5 as the acceptor for the kinetic FRET assay so that the folded (docked) conformation (distance between 3′ and 5′ ends: ∼3 nm) of the HpRz exhibits a higher FRET signal compared with the undocked state (distance between 3′ and 5′ ends: ∼8 nm).

### PAGE analysis of the self-cleavage reaction

The ambient pressure reactions were carried out in a thermoshaker and the HHP reactions in a home-built high-pressure vessel. The reaction products were analysed by denaturing urea polyacrylamide gel electrophoresis (urea PAGE) using a XCell SureLock Mini-Cell system (ThermoFisher) with Novex 15% TBE-Urea gels (ThermoFisher) and TBE running buffer (Ambion). After electrophoresis, the gels were directly stained with SYBR Gold (Molecular Probes) and the bands were analysed by an ultraviolet transilluminator (AlphaImager Mini, ProteinSimple). The intensity of each RNA fragment band was quantified using ImageJ software (N.I.H., USA) and the reaction product is presented by the percentage of cleaved RNA over the total RNA amount (for details, see the Supplementary Information). Hence, the PAGE results take into account the native and cleaved states of the HpRz, only, and, in stark contrast to the FRET data, do not contain any information on undocking conformations and/or dissociation steps.

### Simulations and mod-HpRz

Our simulation studies are based on the crystal structure^29^ of the HpRz in the docked state, which is a modified version of the aforementioned wt-HpRz. This crystal structure contains four dissociated chains. To be able to carry out the experimental studies, we generated the aforementioned mod-HpRz species by taking all the available nucleotides from the crystal structures while adding the missing ones from wt-HpRz^19^. As pointed out earlier^4^, only essential nucleotides, which are identical in wt-HpRz, FRET-HpRz, and mod-HpRz, are required for the catalytic activity of the HpRz. Divalent ions, such as Mg^2+^, strongly stabilize the catalytically active HpRz structure in its docked conformation, however, do not participate directly in the reaction chemistry^53^,^54^. It has also been shown that cobalt(III)hexammine (which was in the crystal structure^29^) cannot directly interact with the reaction site and, moreover, that high concentrations of monovalent salts (Na^+^, Li^+^) can fully support folding as well as catalysis of the HpRz^55^. This is demonstrated by Supplementary Fig. 1 where the cleavage rate of mod-HpRz in the presence of Co(III) is compared with that in the presence of MgCl_2_ at ambient and high pressures. In conclusion, therefore, mod-HpRz without divalent cations at the reaction center as in our simulated model system operates in a very similar fashion as wt-HpRz.

### High-pressure FRET spectroscopy

In all measurements, the FRET signal between the two fluorescent dyes Cy3 and Cy5 was recorded with a K2 fluorescence spectrometer from ISS, Inc., Champaign, IL, USA. The spectrometer uses a xenon arc lamp as light source. The Cy3 fluorescent dye was excited at 550 nm and the real time FRET signal between Cy3 and Cy5 was followed at 677 nm. Cy3 and Cy5 are the most widely used fluorescent sulfoindocyanines for covalently labelling nucleic acids due to their remarkable stability against photobleaching. The Förster distance of the Cy3-Cy5 pair is *R*_0_≈6 nm (ref. 56). In all experiments, the temperature was controlled with a circulating water bath to an accuracy of 0.1 °C, and it was given sufficient time to equilibrate the sample chamber. In case of the pressure dependent measurements, a high-pressure cell with quartz windows was used^57^. The pressure was controlled with an advanced pressure products automated pressure control system (APCS, Ithaca, NY). Reaction mixtures equilibrated at a given temperature were subjected to constant hydrostatic pressures ranging from 1 bar to 2 kbar. The real time FRET signal was recorded at various time scales (up to 1.5 h). The total assembling of the high-pressure cell and pressure system took about 8 min. Therefore, the ribozyme's cleavage reaction had already started, which was taken into account in the data analysis. We observed that with increasing pressure, the solo fluorescence intensity of both dyes, Cy3 and Cy5, increased, in good agreement with previous studies, showing that the fluorescence emission of cyanine dyes increases with increased solution viscosity^57^. The application of pressure increases the viscosity of the solution which leads to an increase of the barrier to bond rotation in the excited state of cyanine dyes, resulting in a decrease of the efficiency of *trans*→*cis* isomerization from the first excited state, which is responsible for the low quantum yield of the fluorophore^57^. The acceptor Cy5 fluorescent dye intensity displays a complex behavior, highly dependent on temperature and pressure. That is why the intrinsic fluorescence emission of Cy5 as a reference measurement was recorded separately along with every single measurement and was corrected in the calculation of the FRET signal. The FRET signal, *I*(*p,T*), of the acceptor Cy5 was determined as a function of temperature and pressure, and, after correction for its intrinsic temperature and pressure dependence (as *I*(*p*,*T*)/*I*_intr_(*p*,*T*)), normalized to the maximum intensity recorded, that is, *I*(10 °C, 1 bar, 6 mM Mg^2+^)=1. In this way, the relative changes of the FRET intensity and, hence, distance information could be obtained as a function of temperature and pressure (for more details see Supplementary Note 2 and Supplementary Fig. 5).

### HPSF FRET measurements

To recover also the initial events of RNA HpRz docking and the cleavage reaction, we carried out complementary measurements of the fluorescence intensity using a HPSF system (HPSF-56 of Hi-Tech Scientific). The real time FRET signal was measured at exactly the same conditions (0.5 μM final RNA HpRz concentration in 50 mM Tris/HCl, 0.1 mM EDTA cleavage buffer at 10 °C with 6 mM Mg^2+^), at 1 bar, 1 kbar, and 2 kbar from 0 up to 500 s. The FRET signal was recorded from the moment of mixing RNA HpRz diluted in cleavage buffer with cleavage buffer solution containing 6 mM Mg^2+^. Along with each experiment, the intrinsic fluorescence emission of Cy5 as a reference measurement was recorded and was used to correct the time-lapse FRET signal to visualize only the catalytic activity of the RNA HpRz.

### Simulation methods

Extensive large-scale replica-exchange molecular dynamics simulations were carried out on the AP state (based on crystal structure PDB ID: 2OUE^29^) of the mod-HpRz ribozyme (see above for sequence) using Version 5.0.2 of the Gromacs simulation package^58^ in the *NpT* ensemble together with the all-atom ff99bsc0χ_OL3_ force field^37^,^38^. This particular force field variant is based on the usual Amber 99 force field with corrections to α- and γ-torsions as well as to glycosidic bond torsions χ. The AP structure underlying our simulation is the docked state of the ribozyme where O2' of A-1 is deprotonated, the scissile phosphate is protonated at its pro-R oxygen, and the A38 nucleobase is protonated at N1 (see Fig. 2b and below for more details) as elaborated in ref. 33. The force field parameters were adjusted for AP by redefining the changed atom types. This model ribozyme was explicitly solvated using the TIP4P/2005 water model^59^, which has been shown to perform well also at high-pressure conditions including the solvation of biomolecules in aqueous solutions at 10 kbar as investigated in ref. 60. An orthorhombic box was chosen such that no ribozyme atom was <15 Å apart from any of the box edges. Ions (Na^+^ and Cl^−^) were added to neutralize the negative charge on the ribozyme and to attain a salt concentration of 0.14 M to mimic experimental conditions. The optimized parameters for explicit biomolecular simulations were taken for these Na^+^ and Cl^−^ ions^61^. The resulting system consisted of approximately 44,265 atoms (1,965 ribozyme atoms, 94 Na^+^ ions, 38 Cl^−^ ions and 14,056 water molecules). Analysis of the simulated structures with respect to the experimental crystal structure obtained at ambient pressure conditions in terms of root mean square deviation (RMSD) (Supplementary Fig. 6) shows that the force field set-up underlying our study is sufficiently reliable in order to keep the ribozyme close to the relevant AP state given our sampling statistics as described below (whereas no attempt whatsoever was made to exhaustively explore the conformational space as described by the particular state-of-the-art RNA force field that we use).

The simulations employed the leap-frog algorithm with a time step of 2 fs. Nosé-Hoover thermostatting^62^,^63^ and Parrinello-Rahman barostatting^64^ with time constants of 1.5 and 1.0 ps, respectively, was used to generate constant temperature and pressure conditions. Altogether with periodic boundary conditions, the smooth Particle-Mesh Ewald method^65^ was applied to treat electrostatic interactions with real space cutoff of 12 Å. A cutoff distance of 12 Å was used for van der Waals interactions. All covalent bond distances involving hydrogen were constrained using the LINCS algorithm^66^, whereas all other degrees of freedom were kept dynamical in all production runs. The simulations were performed independently at 1 bar and at an elevated pressure of 10 kbar in the *NpT* ensemble. In order to compress the system up to a hydrostatic pressure of 10 kbar, the pressure was increased in steps of 400 bar using 25 steps, while the systems were equilibrated for 500 ps at each such step (see Supplementary Note 3 for more details).

### Enhanced sampling

The temperature replica-exchange molecular dynamics (T-REMD) technique^67^,^68^ has been used in order to sample the configuration space of mod-HpRz in its AP state at 300 K and 1 bar as well as at 10 kbar. Such a high pressure has been chosen in order to probe convincingly the structural and energetic changes due to applying HHP conditions being relevant to the kbar regime in view of the well-known pronounced dynamical flexibility of such systems and the resulting strong fluctuation effects on their properties. Each of these T-REMD simulations consisted of as many as 64 replica in the temperature range from 300 to 404 K. The temperature for each replica was generated using the predictor available at http://folding.bmc.uu.se/remd/, which implements the procedure that has been introduced in ref. 69. All replica were equilibrated for 1 ns at the corresponding temperature and at pressures of 1 bar and 10 kbar. The exchange of coordinates was attempted every 2 ps with a probability^70^ given by


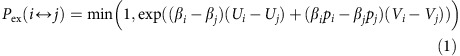


where *β*_*i*_=1/*k*_B_*T*_*i*_, *T*_*i*_, *U*_*i*_, *p*_*i*_ and *V*_*i*_ is the temperature, potential energy, pressure and volume of the system in the state *i*. The obtained exchange rates for the replica ranged from about 20 to 30%. Each system was simulated for 200 ns independently at 1 bar and 10 kbar, hence amounting to ∼25.6 μs of total simulation time. The validity of the exchange sampling was checked based on the potential energy and volume distributions for all the replica, where a significant overlap between the adjacent replica was observed. We also checked that each replica visited all the temperatures at least once during the simulation time. Last but not least, we checked the quality of the resulting sampling directly at the level of the generated distribution functions shown in Figs 5, 6, 7 by only considering the second half of the T-REMD trajectories and confirmed the quality of the presented data; in particular the minimum free-energy pathways at ambient and high pressure agree nearly quantitatively with the ones obtained using the full statistics in Fig. 6 and the hydrogen bond distribution functions reproduce the same trend upon compression as shown in Fig. 7.

### Data availability

Data supporting the findings of this study are available within the article and its Supplementary Information Files. All other relevant data supporting the findings of this study are available on request.

## Additional information

**How to cite this article:** Schuabb, C. *et al*. Pressure modulates the self-cleavage step of the hairpin ribozyme. *Nat. Commun.* **8,** 14661 doi: 10.1038/ncomms14661 (2017).

**Publisher's note:** Springer Nature remains neutral with regard to jurisdictional claims in published maps and institutional affiliations.

## Supplementary Material

Supplementary Information Supplementary Figures, Supplementary Notes and Supplementary References.
