# Supplementary material for: Pressure modulates the self-cleavage step of the hairpin ribozyme
Source: Nat Commun. 2017 Mar 30;8:14661. doi: 10.1038/ncomms14661 (PMC5379106; doi:10.1038/ncomms14661)
Supplement: Supplementary Information — Supplementary Figures, Supplementary Notes and Supplementary References. [file ncomms14661-s1.pdf]

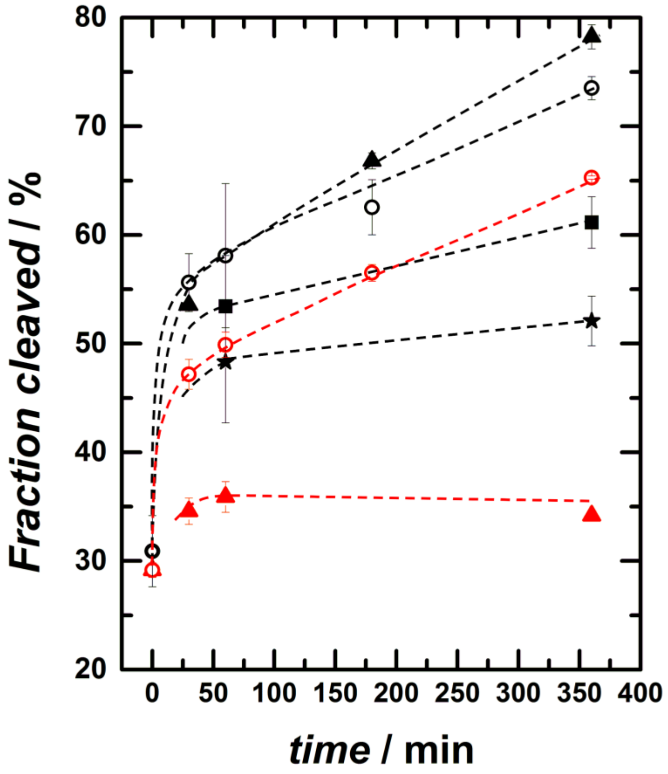

Supplementary Figure 1. **Pressure dependence of the self-cleavage reaction of the modified hairpin ribozyme.** Time evolution of the modified hairpin ribozyme's fraction cleaved for the reaction in the presence of cobalt(III)hexammine (black) or $MgCl_2$ (red) (50 mM Tris-HCl buffer + 0.1 mM EDTA, pH 7.5, 12 mM cobalt(III)hexammine and 100 mM NaCl or 6 mM $MgCl_2$) (mean values. of three independent measurements ± s.d.). Atmospheric pressure (open circles), 1 kbar (closed triangles), 2 kbar (closed squares) and 3 kbar (closed stars).

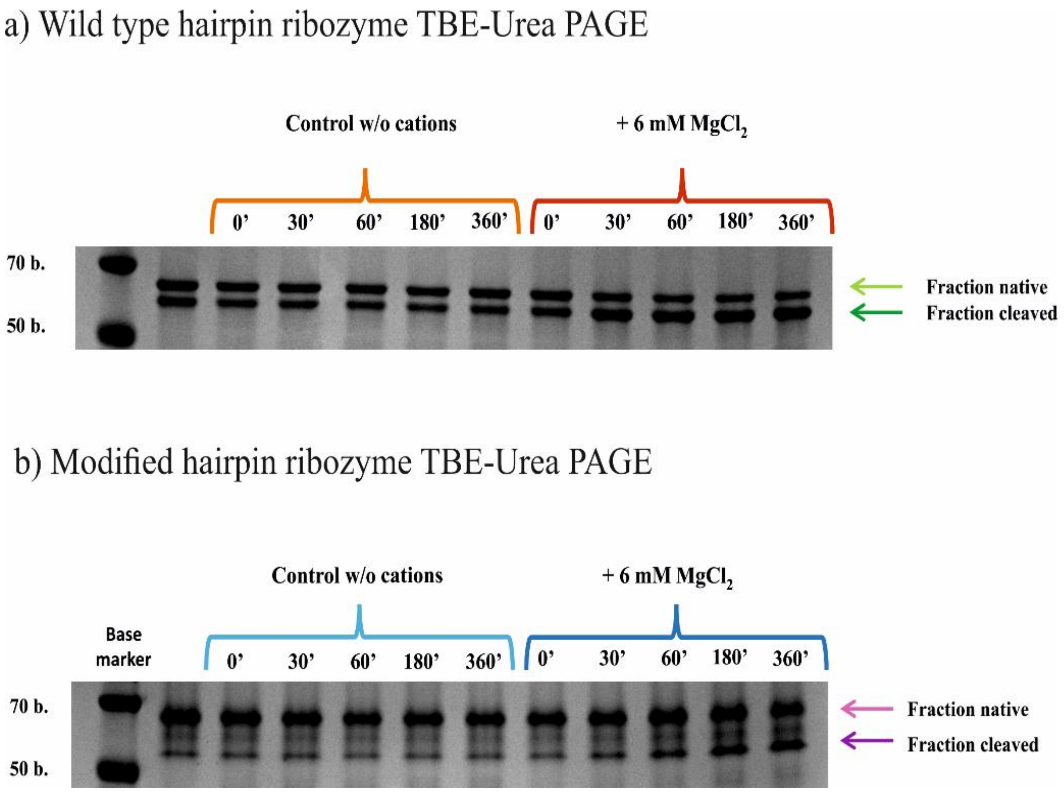

Supplementary Figure 2. Urea denaturing polyacrylamide gel electrophoresis (PAGE) on the overall cleavage reaction. a) TBE-Urea PAGE example of the wild type hairpin ribozyme self cleavage reaction in pure 50 mM Tris buffer + 0.1 mM EDTA pH 7.5 (orange) and in Tris buffer + 0.1 mM EDTA + 6 mM $MgCl_2$ pH 7.5 (red), during 6 h at 25 ^oC. b) TBE-Urea PAGE example of the modified hairpin ribozyme self cleavage reaction in pure 50 mM Tris buffer + 0.1 mM EDTA pH 7.5 (light blue) and in Tris buffer + 0.1 mM EDTA + 6 mM $MgCl_2$ pH 7.5 (dark blue), during 6 h at 25 ^oC.

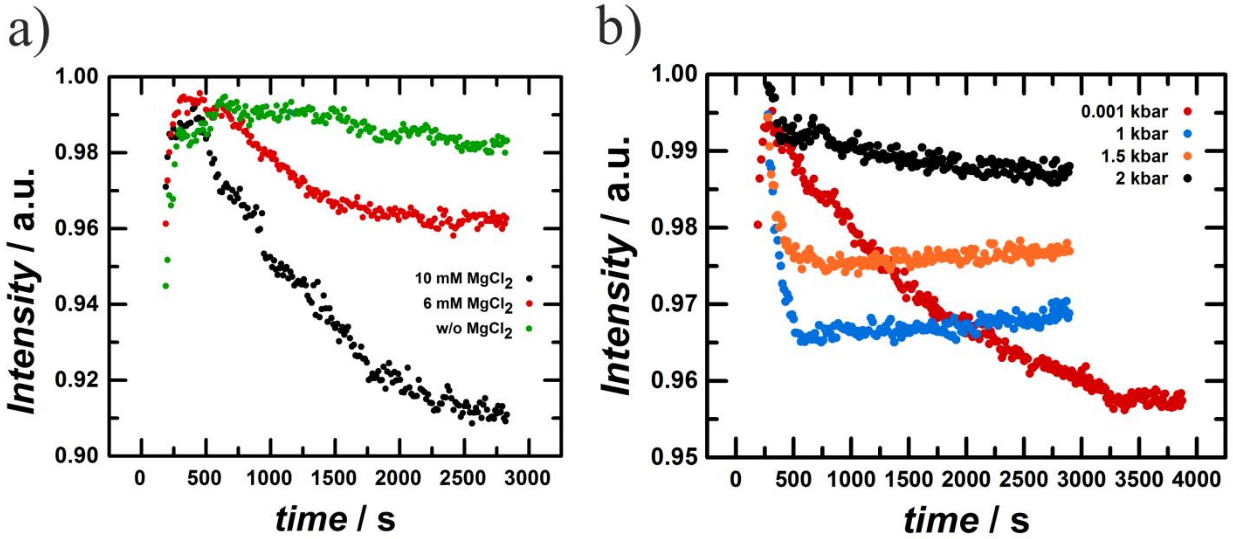

Supplementary Figure 3. **Effect of ionic strength and pressure on the docking/cleavage reaction.** a) FRET data of the Cy3/Cy5 fluorescently labeled HpRz at 10 ^{o}C without Mg^{2+} (green), with 6 mM Mg^{2+} (red) and 10 mM Mg^{2+} (black) (0.5 µM HpRz, 50 mM Tris, 0.1 mM EDTA buffer, pH 7.5). An increased concentration of Mg^{2+} fosters self-cleavage of the HpRz, as the docked state is stabilized in the presence of divalent ions. The initial rise of the FRET signal is due to the docking reaction, the subsequent decay due to the cleavage reaction. b) Pressure dependent FRET data of the HpRz at 10 ^{o}C with 6 mM Mg^{2+} at ambient (red), 1 kbar (blue) 1.5 kbar (orange) and 2 kbar (black). The dead time necessary for sample preparation and loading of the high-pressure cell was ~ 8 min; hence, the initial docking process of the HpRz is absent in the pressure dependent data.

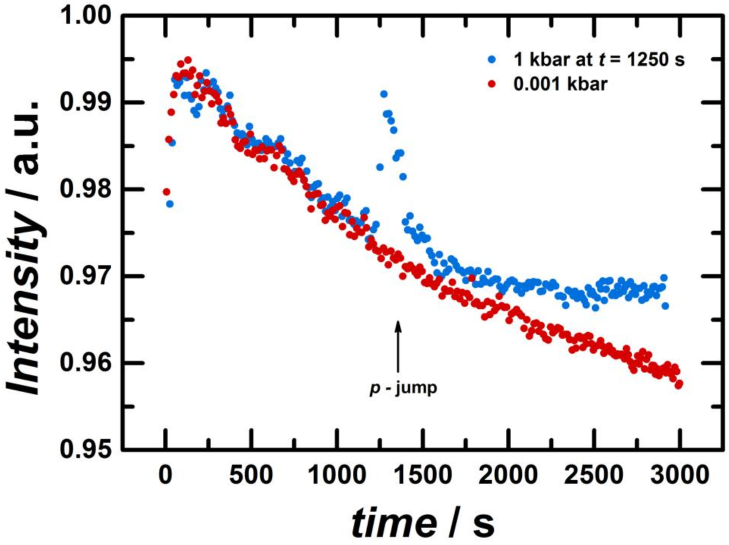

Supplementary Figure 4. **Effect of a pressure-jump on the docking/cleavage reaction.** Time-dependent FRET data of the RNA hairpin ribozyme at 10 °C with 6 mM Mg^{2+}. Triangular data (red) correspond to ambient pressure conditions (1 bar). In a second run, a 1 kbar pressure-jump was applied at a time point of 1250 s, and the FRET intensity was recorded up to about 3000 s (blue). 0.5 µM RNA Ribozyme labeled with Cy3' and Cy5' fluorescent dyes at loop A and loop B was measured in 50 mM Tris and 0.1 mM EDTA (pH 7.5).

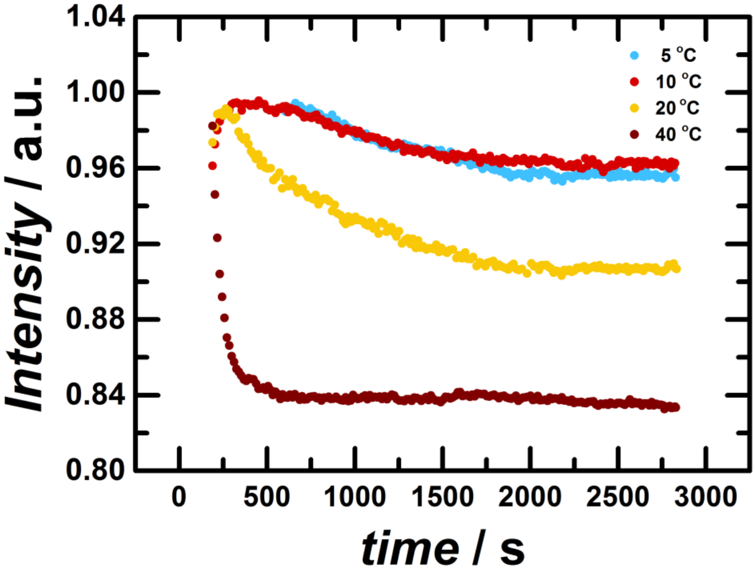

Supplementary Figure 5. **Effect of temperature on the docking/cleavage reaction.** FRET data of the RNA hairpin ribozyme at 5 °C (blue), 10 °C (red), 20 °C (yellow) and 40 °C (brown) in the presence of 6 mM Mg^{2+}. 0.5 µM RNA ribozyme labeled with Cy3' and Cy5' fluorescent dyes at loop A and loop B was measured in 50 mM Tris and 0.1 mM EDTA buffer (pH 7.5). With increasing temperature, from 5 to 40 °C, the cleavage process is drastically accelerated, and the docking reaction is hardly observable anymore already at 20 °C under these conditions. Therefore, we chose the temperature of 10 °C for our experimental studies.

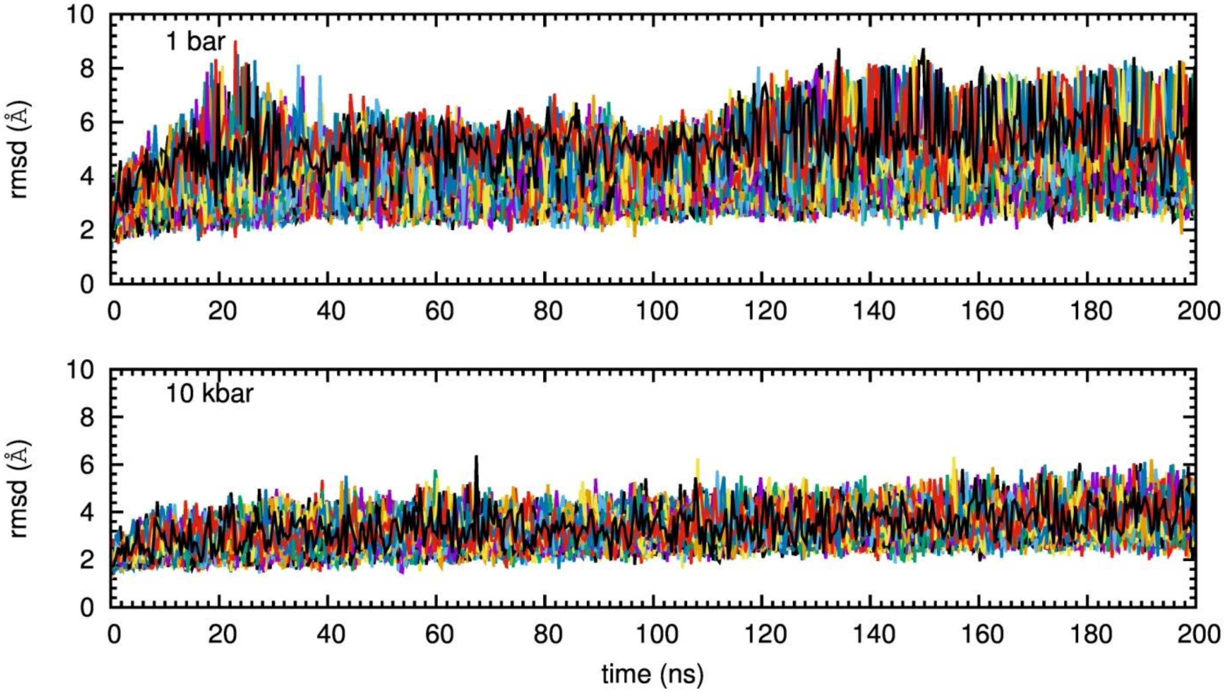

Supplementary Figure 6. Root mean square deviation (RMSD) at a function of T-REMD simulation time of all heavy atoms of each of the 64 replica (which cover the temperature range from 300 to 404 K encoded used different colors) of the hairpin ribozyme w.r.t. its crystal structure (PDB ID: 2OUE from Ref.[1]). The RMSD shows that the AP structure of the ribozyme does not break apart neither at ambient conditions nor upon significant compression according to the particular force field even when increasing the temperature considerably beyond ambient to enhance sampling via T-REMD. Moreover, this force field supports significant fluctuations of individual replica around the AP state. This behavior allows us to map out the free energy landscape in the subspace that is relevant to the self-cleavage reaction within the AP state as depicted in Figs. 5 and 6 of the main text as well as to compute the structural distributions functions shown in Fig. 7.

Supplementary Note 1

PAGE Analysis of the Self-cleavage Reaction. All materials and reagents related with sample handling were nuclease free (molecular biology grade) or cleaned with RNase AWAY® reagent (Molecular BioProducts), and then rinsed with nuclease free water (Omega Bio-tek, Inc.). Buffers were filtered with Whatman® 0.2 µm (cellulose acetate membrane) sterile filter. The high hydrostatic pressure reactions were carried out in a home-built high-pressure vessel connected to a manual pressure pump, using water as a pressure-transmitting medium, and the temperature was controlled by an external water bath. The RNA was stored at -80 ^{o}C in pure deionized water. Prior to the experiments, samples were aliquoted and lyophilized. The lyophilized ribozyme samples were then dissolved in 50 mM Tris-HCl buffer, pH 7.5, with 0.1 mM EDTA, at a final concentration of 28.7 µM. These samples were subjected to denaturation and renaturation steps to ensure proper folding (heating up to 90 ^{o}C for 1 min, subsequent cooling down to 25 ^{o}C at a rate of 3 ^{o}C min^{-1}). The self-cleavage reaction started (t = 0) by mixing the RNA solution 1:1 with 12 mM $MgCl_2$ or 24 mM $[Co(NH_3)_6]Cl_3$ + 200 mM NaCl buffer solution (deadtime for the ambient pressure measurements: 20 s). All reactions were conducted at 25 ^{o}C, the time varied from 0 to 360 min and the pressure range covered was from 1 bar to 3 kbar (deadtime for the pressure-dependent measurements: 4 min). The ambient pressure reactions were carried out in thermoshaker (cooling thermal shake touch, VWR). The high hydrostatic pressure reactions were carried out in a home-built high-pressure vessel connected to a manual hydraulic pump using water as pressure-transmitting medium. The temperature was controlled by an external water bath. The samples were placed in home-built high pressure Teflon bottles and sealed with pressure-transmitting Dura Seal film fixed by a rubber O-ring. Each data point was measured with a freshly prepared new sample. The ribozyme's self-cleavage reaction was stopped by adding 1:1 stopping buffer (7 M urea + 50 mM EDTA, pH 7.5), with the further addition of RNA loading buffer 2X (AMRESCO®), and stored in ice. Final aliquots of 10 µL were stored at -80 ^{o}C.

The reaction products were analyzed by denaturing Urea Polyacrylamide Gel Electrophoresis (Urea PAGE) using the XCell SureLock® Mini-Cell system (ThermoFisher) with Novex® 15% TBE-Urea gels (ThermoFisher) and TBE running buffer (Ambion®). Cleavage reaction's aliquots of 10 µL containing 3.6 µM of RNA were applied to the gels. After electrophoresis, the gels were directly stained with SYBR® Gold (Molecular Probes™), and the bands were analyzed by an ultraviolet (UV) transilluminator (AlphaImager Mini, ProteinSimple). The light intensity of each RNA fragment band was quantified using ImageJ software (N.I.H., USA),[2,3] and the reaction product is presented by the percentage of cleaved RNA over the total RNA amount (cleaved RNA + native RNA) (Supplementary Fig. 3 shows an example).

Supplementary Note 2

Sample Preparation for the FRET Measurements. The RNA hairpin ribozyme was stored in a lyophilized state and was diluted freshly before the measurements in a cleavage buffer consisting of 50 mM Tris/HCl and 0.1 mM ethylenediaminetetraacetic acid (EDTA), pH 7.5. The final concentration of RNA hairpin ribozyme used was 0.5 µM in all FRET measurements. The RNA was subjected to denaturation and renaturation steps (heated to 90 ^{o}C for 1 min and slowly cooled till 25 ^{o}C in 3 ^{o}C min^{-1} steps) to ensure proper folding of the RNA loops. The cleavage reaction started when lyophilized samples of RNA hairpin ribozyme was diluted into the cleavage buffer containing various concentrations of Mg^{2+} ions. Measurements were performed at various Mg^{2+} concentration (6 mM, 10 mM), temperature (5, 10,

20, and 40 ^oC) (Supplementary Fig. 2), and pressure (1 bar, 1.0, 1.5, and 2.0 kbar) conditions as indicated. As the cleavage reaction increases rapidly with increased temperature and Mg^{2+} concentration, to slow down the reaction rate and being able to observe also the initial steps with sufficient time resolution, we chose a temperature of 10 ^oC and a low Mg^{2+} concentration of 6 mM for the high pressure measurements, as mounting and closing the pressure cell requires some time (dead time 3-8 min).

Supplementary Note 3

Relaxation and Thermalization. The following multistep relaxation procedure has been applied to equilibrate the system. First, the system was energy minimized using standard steepest-descent for 2000 steps in vacuum followed by 10000 steps of steepest-descent energy minimization after solvating the ribozyme with explicit water and ions. After that, 2 ns of simulated annealing was carried out from 50 to 400 K in the *NVT* ensemble with position restraints acting on all heavy atoms including the water oxygen sites. The system was heated from 50 to 400 K at the rate of 0.5 K/ps and thereafter the system was kept at 400 K for 500 ps. After that, it was annealed down to 300 K with the same rate and was kept at 300 K for another 600 ps. After the annealing stage, the system was again energy minimized for 10000 steps followed by 500 ps of simulation in the *NVT* ensemble. Next, 500 ps of simulation was performed in the *NpT* ensemble at p = 1 bar and T = 300 K subject to the aforementioned heavy-atoms restraints.

Supplementary References:

1. Salter, J., Krucinska, J., Alam, S., Grum-Tokars, V. & Wedekind, J. E. Water in the active site of an all-RNA hairpin ribozyme and effects of Gua8 base variants on the geometry of phosphoryl transfer. *Biochemistry* **45,** 686–700 (2006).
2. Schneider, C. a, Rasband, W. S. & Eliceiri, K. W. NIH Image to ImageJ: 25 years of image analysis. *Nat. Methods* **9,** 671–675 (2012).
3. Rasband, W. *ImageJ 1997-2007. U. S. Natl. Institutes Heal. Bethesda, Maryland, USA* (2008).
